# Prevalence of nasal colonization by methicillin-resistant *Staphylococcus aureus* in outpatients living with HIV/AIDS in a Referential Hospital of the Northeast of Brazil

**DOI:** 10.1186/s13104-018-3899-z

**Published:** 2018-11-06

**Authors:** Cynthia Regina Pedrosa Soares, Celso Rodrigues de Lira, Maximiliano Alexandre H. Cunha, Valter Romão de Souza Junior, Fábio Lopes de Melo, Paulo Sergio Ramos de Araújo, Maria Amélia Vieira Maciel

**Affiliations:** 1 Department of Tropical Medicine, Federal University of Pernambuco, Av. Prof. Moraes Rego 1235, 5067-0901 Recife, Brazil; 2 Fundação Oswaldo Cruz-Fiocruz, Instituto Ageu Magalhaes, Avenida Professor Moraes Rego, s/n Cidade Universitaria, Recife, 50670-420 PE Brazil; 3 Universidade Federal de Pernambuco, Av. Prof. Moraes Rego 1235, 5067-0901 Recife, Pernambuco Brazil

**Keywords:** *Staphylococcus aureus*, MRSA, CA-MRSA, HIV, mecA

## Abstract

**Objective:**

The purpose of this study is to investigate the prevalence of MRSA among people living with HIV/AIDS (PLHA) being monitored in a tertiary outpatient hospital in the state of Pernambuco, in the Brazilian Northeast.

**Results:**

*Staphylococcus aureus* was isolated from a nasal swab and found in 31.4% of the individuals (95% CI 27.3–35.5), of whom 4.4% (95% CI 8.5–19.5) were MRSA, as confirmed by the presence of the mecA gene. For individuals whose *S. aureus* was recovered, the mean age was 41.5 years; 93.6% were on antiretroviral treatment. This group had CD4 cell counts > 200 (92%) and viral load ≤ 100 copies (79.1%). Use of antimicrobial agents in the past 12 months was found among 21% of the individuals, and 24.2% reported use of illicit drugs at lease once in their lifetime. Prevalence of nasal colonization by MSSA (26.7%) and MRSA (4.4%) was higher in comparison to other studies of this population; nevertheless, we were unable to establish factors associated with risk.

**Electronic supplementary material:**

The online version of this article (10.1186/s13104-018-3899-z) contains supplementary material, which is available to authorized users.

## Introduction

Methicillin-resistant *Staphylococcus aureus* (MRSA) is considered a pathogen of great relevance because of its distribution in hospital epidemiology. It is related to exposure and hospital stay, as well as the use of invasive apparatuses [1]. The frequency of colonization or infection by MRSA in individuals outside of the hospital environment, classified as community-acquired methicillin-resistant *Staphylococcus aureus* (CA-MRSA), has progressively increased in recent decades; and this tendency has likewise been observed in people living with HIV/AIDS (PLHA). In fact, among them this seems to be even greater than in the population without HIV [2, 3].

The nasal colonization rate in PLWHA in Taiwan was 6% [4]; in Iran, 5.3% [5]; in India, 16.6% [6]; and in Dallas, USA, 10.8% [7]. There are few publications concerning the prevalence of MRSA in the Brazilian population. In São Paulo, one study found a prevalence of 21.7% [8, 9].

The aim of this study was to investigate the prevalence of nasal colonization by MRSA and associated risk factors among PLHA attended in the outpatient care unit of the University Hospital of Pernambuco.

## Main text

### Methods

A cross-sectional study of an analytical nature, to detect the prevalence of nasal colonization by MRSA and establish associations with immunological and viral status and prior use of antimicrobial agents among PLHA attended in the outpatient unit of the Clinical Hospital of the Federal University of Pernambuco (HC-UFPE) in Pernambuco Brazil. Sociodemographic information (Table 2), as well as the epidemiological clinic (Table 3 and Additional file 1, respectively) were captured through patients medical records.

#### Collection of nasal swab samples

All PLHA from HC-UFPE were invited to participate of this study. Nasal secretion samples were collected from all PLHA over 18 years of age, who agreed to participate in the study by signing the Free and Informed Consent Form (ICF). Care was taken to ensure that no patient was enrolled twice during the study. The nasal swab samples were taken from PLHA attended in the outpatient clinic of the Infectious and Parasitic Disease Service, during the period 2 years, using swab in a Stuart transportation medium.

#### Isolation and identification of *Staphylococcus*

Colonies with the morphological characteristics of the genus *Staphylococcus aureus* were submitted to identification tests, using DNAse and Staphlin^®^ catalysis. Only those samples with characteristics of *S. aureus* were referred for presumptive and molecular testing for detection of resistance to methicillin.

#### Test for cefinase-β-lactamase

All the isolates identified as *S. aureus* were submitted to the cefinase test; 138 (88%) of them were positive, carriers of β-lactamase enzymes.

#### Extraction of DNA from the isolates of *S. aureus* by the polymerase chain reaction (PCR)

Extraction of deoxyribonucleic acid (DNA from the MRSA samples was performed using the phenol–chloroform technique, in which one 1 mL aliquot of bacterial culture previously grown in a BHI solution at 37º for 24 h was centrifuged at 14,000 rpm/4 °C. Using the phenol–chloroform method, its pellet was subjected to extraction of DNA.

#### Determination of type of SCCmec

All positive isolates for research on the gene mecA were submitted to determination of the type of SCCmec, performed utilizing the Multiplex-PCR method, as per the protocol of Kondo et al. [10]. The PCR reactions were prepared in 50 µL volume in each tube, following the protocol for complex ccr, utilizing primers mA1, mA2, α1, α2, α3, γF and γR. The products of the amplification were subjected to electrophoresis in a 2% agarose gel, stained blue-green (LGC Biotechnology), using a 100 pb molecular weight marker from Invitrogen, generating fragments of different sizes, which were analyzed by the Kodak 1D version 3.5.2 software (Scientific Imaging Systems, USA).

#### DNA sequencing of MRSA samples

DNA sequencing reactions were carried out at the Nucleus of Technological Platforms (NPT) of the Aggeu Magalhães-Fiocruz-PE Research Center, using the ABI 3500 × L Genetic Analyzer (Applied Biosystems).

The commercially available ABI PRISM BigDye Terminator Cycle Sequencing v 3.1 Ready Reaction Kit (Applied Biosystems^®^) was used according to the manufacturer’s recommendations. The products of the reactions were analyzed in the ABI 3500 × L Genetic Analyzer sequencer, developed by Applied Biosystems.

For the reaction, 1 μL primer (3.2 pmol/μL), 0.5 μL Bigdye^®^ Terminator v3.1, 1 μL 5 × sequencing buffer (Tris–HCl, pH 9.0 200 mM, 5 mM MgCl_2_), 1 μL of the purified PCR product (10 ng/μL) and MilliQ water 10 μL q.s.p. The amplification program followed the following cycling: 1 cycle at 94 °C for 2 min, followed by 40 cycles of 94 °C for 15 s, 50 °C for 10 s and 60 °C for 4 min. For each sample, two reactions were performed, one for the forward primer and the other for the reverse. After amplification, DNA purification/precipitation was performed: to the total volume of the sequencing reaction (10 μL) were added 2.5 μL of EDTA (125 mM, pH 8.0) and 25 μL of 100% ethanol. After vortexing, the reaction was centrifuged at 3700 rpm for 45 min. The reaction precipitate was washed with 70 μL of 70% ethanol, followed by centrifugation at 3700 rpm for 10 min. After discarding the supernatant, the precipitate was solubilized in 10 μL Formamide Hi-Di (Applied Biosystems) and applied to the automated sequencer.

### Statistical analysis

The data gathered were analyzed using version 6.04 of the EpiInfo software. Wherever appropriate, statistical comparisons were made using the odds ratio. The value p < 0.05 was considered a statistically significant difference.

### Results

**Fig. 1:**
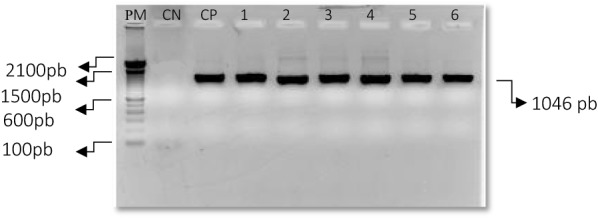
Representation of the PCR amplification product of the mecA region of *S. aureus* in outpatient isolates of PLHA. PM, molecular weight; CN, negative control; CP, positive control; 1–6 DNA extracted from patients

Participating in the study were 500 PLHA, of whom 157 were *S. aureus* positive using nasal swab; this corresponds to a prevalence of 31.4% (95% CI 27.3–35.5). Among the *S. aureus* positives, positivity of MRSA was 14% (95% CI 8.5–19.5). Therefore, the prevalence of MRSA in PLHA was 22/500 (4.4%). Only those samples showing resistance by methicillin screening were confirmed by detection of the mecA resistance gene, using the PCR technique (Fig. 1). The PCR multiplex showed the amplification of the class A ccr complex. All the isolates presented genes ccrA1, ccrA2 and ccrA3, indicating types I, II and III, respectively. Like majority of the isolates, 12/22 presented the most essential gene of the SCCmec complex, the mecA gene, indicting type II SCCmec. The ccrC gene was found in 7/22 isolates—which indicates type V of SCCmec.

#### Analysis of the data on DNA sequencing of the isolates of *S. aureus*

The sequencing of the DNA of the mecA region was performed and compared with the sequence of the Locus KR936061 and KR9360059 of the BLAST. Thus, 100% similarity was proven. Online alignment was done by the Mafft, in accordance with the “Mafft” algorithm.

The sequencing data were generated by the Standn Package (Console, Gap 4, Pregap 4, Spin and Trev); subsequently, they were analyzed in BLAST and two sequences of the region mecA of *S. aureus* to compare the consensus identity with the database.

#### Profile of susceptibility of the MRSA isolates

Most of the MRSA were classified as multi-resistant, showing resistance to more than two classes of antibiotics. The profile of antimicrobial sustainability of the isolates of *S. aureus* was determined by the fusion disk method.

Considering the profile of antimicrobial susceptibility of the patients colonized by MRSA of the antimicrobial classes tested, greatest resistance was found to cefoxitin (73%), erythromycin (68.2%) and sulfamethoxazole–trimethoprim (68.2%). As for penicillin, all patients showed resistance (Table 1). Induced resistance to clindamycin, by D test, was observed in 31% of the MRSA.

**Table 1:** Antimicrobial susceptibility profile of MRSA isolates according to antimicrobial classes in PLHA treated at the HC/UFPE DIP service

Samples	Antimicrobial resistance
SN 65	ERI, CLI, PEN
SN 109	CFO, CLO, GEN, ERI, CLI, PEN, SUT
SN 111	ERI, PEN, SUT
SN 133	CFO, CLO, CLI, PEN
SN 170	CFO, CLO, CIP, GEN, ERI, CLIN, PEN, SUT
SN 172	CFO, GEN, ERI, CLI, PEN, SUT
SN 193	CFO, GEN, ERI, CLI, PEN, SUT
SN 211	CFO, PEN
SN 241	CFO, ERI, PEN, SUT
SN 300	CFO, CLO, PEN
SN 333	CFO, CLO, PEN, SUT
SN 334	CFO, CLO, CIP, GEN, CLI, ERI, PEN, SUT
SN 335	CFO, CLI, PEN
SN 338	CFO, CIP, GEN, ERI, CLI, PEN, SUT
SN 407	CFO, CLO, CIP, ERI, PEN, SUT
SN 427	CFO, CLO, CIP, ERI, PEN, SUT
SN 436	CFO, CLO, CIP, GEN, ERI, CLI, PEN, SUT
SN 442	CFO, CIP, GEN, ERI, CLI, PEN, SUT
SN 475	ERI, CLI, PEN, SUT
SN 484	GEN, PEN
SN 487	ERI, CLI, PEN, SUT
SN 500	PEN

CFO, cefoxitin; CLO, chlorafenicol; CIP, cyprofloxacin; GEN, gentamycin, CLI, clindamycin; ERI, erythromycin; PEN, penicillin; SUT, sulfamethoxazole–trimethoprim

The isolates resistant to cefoxitin were subjected to PCR for detection of the gene of resistance mecA, in which the presence of this gene was confirmed.

#### Test for cefinase-β-lactamase

All the isolates identified as *S. aureus* were submitted to the cefinase test; 138 (88%) of them were positive, carriers of β-lactamase enzymes.

#### Factors associated with nasal colonization by MRSA

Nasal swab samples were collected from 500 PLHA for a period of 2 years, to evaluate the risk factors associated with colonization by MRSA. *Staphylococcus aureus* were positive in 157 out of 500 samples. Out of them, 22 (4.4%) were resistant to methicillin.

In this population of PLHA, the majority were men, feoderm, with an average age of 41.5 years and with a family income below a minimum wage. There was no significant association between these variables studied and nasal colonization by MRSA, or with habits such as the practice of physical activity, a history of smoking cigarettes, drinking alcoholic beverages, or even having used illicit drugs at once in their lifetime (Table 2).

**Table 2:** Association of MRSA and MSSA positivity according to biological and socioeconomic factors of the PLHA at the HC/UFPE DIP Service

Variables	All patients	Colonization by *Staphylococcus aureus*		OR (IC 95%)	*p* value
		MRSA	MSSA		
Biological					
Sex					
Male	106 (67.5%)	12 (11.3%)	94 (88.7%)	1.0	–
Female	51 (32.5%)	10 (19.6%)	41 (80.4%)	1.91 (0.76–4.77)	0.166
Age					
Mean Â ± SD	41.5 ± 11.5	42.8 ± 11.4	41.3 ± 11.6	1.01 (0.97–1.05)	0.578
Age range					
Less than 40 years	66 (42.0%)	7 (10.6%)	59 (89.4%)	1.0	–
40 years and over	91 (58.0%)	15 (16.5%)	76 (83.5%)	1.66 (0.64–4.34)	0.298
Skin color					
White	41 (26.1%)	8 (19.5%)	33 (80.5%)	1.0	–
Feoderm	79 (50.3%)	11 (13.9%)	68 (86.1%)	0.67 (0.24–1.82)	0.428
Black	37 (23.6%)	37 (8.1%)	34 (91.9%)	0.36 (0.09–1.49)	0.160
Socioeconomic					
Can read and write					
Yes	147 (93.6%)	19 (12.9%)	128 (87.1%)	1.0	–
No	10 (6.4%)	3 (30.0%)	7 (70.0%)	2.89 (0.69–12.1)	0.148
Education					
High school or University	82 (52.2%)	12 (14.6%)	70 (85.4%)	1.0	–
Primary school	47 (29.9%)	3 (6.4%)	44 (93.6%)	1.94 (0.68–5.56)	0.215
Incomplete elementary school	28 (17.8%)	7 (25.0%)	21 (75.0%)	0.40 (0.11–1.49)	0.171
Family income					
Less than 1 minimum wage (MW)	64 (42.4%)	13 (20.3%)	51 (79.7%)	1.0	–
1–2 MW	45 (29.8%)	5 (11.1%)	40 (88.9%)	0.49 (0.16–1.49)	0.209
More than 2 Mw	42 (27.8%)	4 (9.5%)	38 (90.5%)	0.41 (0.12–1.37)	0.148

^a^Statistically significant association (p < 0.05)

Almost all the individuals in this study (93.6%) were under a regime of antiretroviral therapy (ART), and 66% of them manifested defining criteria of AIDS. At the moment of the research, most (92%) had TCD4 cell counts of over 200 cells/mm^3^ and viral load for HIV under 100 copies/mL, but there was no statistically significant association between MRSA nasal colonization and these variables. Although the associations between diagnosis of AIDS and absence of ART were not significant, there was a greater chance of nasal colonization by MRSA in these individuals (Additional file 1).

The use of antibiotics within one year prior to the swab collection was reported by 21% of the individuals studied, but was not associated with the presence of MRSA in nasopharynx (Additional file 1); most of them (83.1%) had received some sulfonamide derivative.

Reports of co-morbidities, such as diabetes mellitus (8.5%), chronic liver disease (7.1%), malignant neoplasms (2.6%) and chronic kidney disease in renal replacement therapy (1.9%); and of prior history of sexually transmitted infection, were not associated with the presence of nasal MRSA (Additional file 2).

### Discussion

*Staphylococcus aureus* is frequently encountered in the nasal mucus of healthy individuals, and may be the agent responsible for community infections and those related to health care. Despite the fact that this pathogen can be found in the nasal mucus of the general population, its incidence has been described as greater in PLHA; few studies have been done in Brazil. The prevalence of *S. aureus* isolated from the nasal mucus of the population studied was 31.4%. The prevalence and incidence of colonization by *S. aureus* varies in accordance with the population studied. A survey of the studies showed that the rate of colonization can reach approximately 38% [11]. The characteristics of each individual, and likewise of health processes, may influence the microbial profile of the hospital and the individual’s mucus. The prevalence of *S. aureus* in our study seems to be similar to worldwide frequencies reported in other studies of the general population, which have varied from 30 to 74% in various regions of the world, particularly in Latin America [12, 13].

The dissemination of MRSA clones may be related to the diversity of colonization of these strains in the world [14]. A typology of the staphylococcal cassette chromosome is essential for an understanding of the molecular epidemiology of methicillin-resistant *Staphylococcus aureus* (MRSA). A multiplex-PCR assay permits the concomitant detection of resistance to methicillin (mecA gene) and the classification of all types and subtypes SCCmec I, II, III, IVa, b, c, d and V, in which 100% sensitivity and specificity in the characterization of 54 strains of MRSA showed that they belong to various types and subtypes of SCCmec [15].

Within the study population, nasal colonization by *S. aureus* and MRSA was more frequent in males. Similar finding was obtained in a study conducted in South Carolina, USA, in which 63% of the sample were men, with mean age of 43.2 years and prevalence of 8% of MRSA [16]. These results corroborate the data from our study regarding sex and age. In our study, the mean age was 41.5 years, mulatto (50.3%). The variables studied were associated with colonization by MRSA, but the correlations were not statistically significant (Table 3). In 2009 [17], Shet et al. presented similar results, where no statistically significant differences were found with regard to age and ethnicity, or schooling, employment situation, exposure to the use of illicit drugs, among the groups studied, with or without HIV. Likewise, in 2015 [18] Farley and collaborators found a prevalence of MRSA nasal colonization of 15.4% in PLHA; most of the sample consisted of black men, but there was no significant association. Nor were sex and age risk factors for nasal colonization by MRSA among PLHA in a study in Iran [5]. These data reinforce the idea that socio-demographic variables are not related to MRSA; thus, there was no association between nasal colonization by MRSA and the variables cited above.

**Table 3:** Association of MRSA and MSSA positivity according to HIV-related habits and HIV-related factors seen at the HC/UFPE DIP Service

Variables	All patients	Colonization by *Staphylococcus aureus*		OR (IC 95%)	p-value
		MRSA	MSSA		
Habits					
Physical activity					
Yes	54 (34.4%)	6 (11.1%)	48 (88.9%)	1.0	–
No	103 (65.6%)	16 (15.5%)	87 (84.5%)	1.47 (0.54–4.00)	0.450
Ethicism					
Never drank	10 (6.4%)	2 (20.0%)	8 (80.0%)	1.0	–
Stylist	56 (35.7%)	9 (16.1%)	47 (83.9%)	0.77 (0.14–4.21)	0.759
Ex-stylist	91 (57.9%)	11 (12.1%)	80 (87.9%)	0.55 (0.10–2.93)	0.484
Smoking					
No	119 (75.8%)	14 (11.8%)	105 (88.2%)	1.0	–
Yes	38 (24.2%)	8 (21.1%)	30 (78.9%)	2.00 (0.78–5.21)	0.156
Illicit drugs					
No	108 (69.2%)	16 (24.8%)	92 (75.2%)	1.0	–
Yes	48 (30.8%)	6 (12.5%)	42 (87.5%)	0.82 (0.30–2.25)	0.702
Related to HIV					
HIV carrier					
Yes	101 (66.4%)	11 (10.9%)	90 (89.1%)	1.0	–
No	51 (33.6%)	11 (21.6%)	40 (78.4%)	2.25 (0.90–5.61)	0.082
Use of Antiretroviral					
Yes	147 (93.6%)	19 (12.9%)	128 (87.1%)	1.0	–
No	10 (6.4%)	3 (30.0%)	7 (70.0%)	2.89 (0.69–12.1)	0.148
Current viral load					
Undetectable	117 (79.1%)	15 (12.8%)	102 (87.2%)	1.0	–
50 a 100,000	29 (19.6%)	6 (20.7%)	78 (79.3%)	1.77 (0.62–5.06)	0.284
> 100,000 copies	2 (1.3%)	0 (–)	16 (100%)	Not Calculated	–
Current CD4					
> 200	138 (92.0%)	20 (14.5%)	118 (85.5%)	1.0	–
< 200	12 (8.0%)	2 (16.7%)	10 (83.3%)	1.18 (0.24–5.78)	0.838
CD4 Nadir					
> 200	94 (62.6%)	13 (13.8%)	81 (86.2%)	1.0	–
< 200	57 (37.4%)	9 (15.8%)	48 (84.2%)	1.17 (0.46–2.94)	0.741
Previous use of antibiotics					
Yes	33 (21.0%)	6 (18.2%)	27 (81.8%)	1.0	–
No	124 (79.0%)	16 (12.9%)	108 (87.1%)	0.67 (0.24–1.86)	0.440

^a^Statistically significant association (p < 0.05)

Our study also sought to evaluate the association between nasal colonization by MRSA and co-morbidities such as diabetes, chronic liver disease, cancer and sexually transmitted diseases, but was unable to establish any significant associations. Hemmige and collaborators [19] observed that none of these variables was associated with nasal colonization by MRSA. A similar study conducted in Iran stressed that there was no association between MRSA nasal colonization and cigarette smoking or prior use of antibiotics [5].

Our findings point to a prevalence of nasal colonization by HA-MRSA among PLHIV similar to that found in the non-HIV population; it was less frequent in those individuals PVHS with prior virologic control and severe immunodepression. However, we were unable to establish correlations with these variables. Future research may be able to determine the role of nasal colonization by CA-MRSA in this population. This study brings the importance of studying more about the epidemiology of MRSA in PLHA in the Brazilian Northeast, covering a set of actions aimed at the detection or prevention of these pathogens, with the purpose of recommending and adopting prevention and control measures.

## Limitations

This study is limited lacking of more detailed information about MRSA carriers: e.g. antibiotic use which is active for MRSA (e.g. TMP/SMZ, doxycycline), live in crowded places, hygiene status, incarceration, HIV transmission risk (MSM, IV drug use), household members hospitalized, multiple sexual partners in prior 12 months, and detailed data of healthcare exposure.

## Additional files

**Additional file 1: Table S1.** Association of MRSA and MSSA positivity according to HIV-related habits and HIV-related factors seen at the HC/UFPE DIP Service.

**Additional file 2: Table S2.** Association of positivity by *Staphylococcus aureus* according to factors related to Comorbidities and co-infections of PLHA treated at an outpatient clinic at the HC/UFPE DIP Service.

## Abbreviations

MRSA: methicillin-resistant *Staphylococcus aureus*
CA-MRSA: community-acquired methicillin-resistant *Staphylococcus aureus*
PLHA: people living with HIV/AIDS
HC-UFPE: Clinical Hospital of the Federal University of Pernambuco
ICF: Informed Consent Form
CLSI: Clinical and Laboratory Standards Institute
