# Supplementary material for: Prevalence of nasal colonization by methicillin-resistant Staphylococcus aureus in outpatients living with HIV/AIDS in a Referential Hospital of the Northeast of Brazil
Source: BMC Res Notes. 2018 Nov 6;11:794. doi: 10.1186/s13104-018-3899-z (PMC6219150; doi:10.1186/s13104-018-3899-z)
Supplement: Supplementary file 1 — Additional file 1: Table S1. Association of MRSA and MSSA positivity according to HIV-related habits and HIV-related factors seen at the HC/UFPE DIP Service. [file 13104_2018_3899_MOESM1_ESM.doc]

Additional file 1. Association of MRSA and MSSA positivity according to HIV-related habits and HIV-related factors seen at the HC / UFPE DIP Service.

| **Variables** | **All patients** | **Colonization by *Staphylococcus aureus*** | | **OR (IC 95%)** | **p-value** |
| --- | --- | --- | --- | --- | --- |
|  |  | **MRSA** | **MSSA** |  |  |
| **Habits** |  |  |  |  |  |
| **Physical activity** |  |  |  |  |  |
| YES | 54 (34,4%) | 6 (11,1%) | 48 (88,9%) | 1,0 | - |
| NO | 103 (65,6%) | 16 (15,5%) | 87 (84,5%) | 1,47 (0,54 – 4,00) | 0,450 |
| **Ethicism** |  |  |  |  |  |
| Never drank | 10 (6,4%) | 2 (20,0%) | 8 (80,0%) | 1,0 | - |
| Stylist | 56 (35,7%) | 9 (16,1%) | 47 (83,9%) | 0,77 (0,14 – 4,21) | 0,759 |
| Ex-stylist | 91 (57,9%) | 11 (12,1%) | 80 (87,9%) | 0,55 (0,10 – 2,93) | 0,484 |
| **Smoking** |  |  |  |  |  |
| NO | 119 (75,8%) | 14 (11,8%) | 105 (88,2%) | 1,0 | - |
| YES | 38 (24,2%) | 8 (21,1%) | 30 (78,9%) | 2,00 (0,78 – 5,21) | 0,156 |
| **Illicit drugs** |  |  |  |  |  |
| NO | 108 (69,2%) | 16 (24,8%) | 92 (75,2%) | 1,0 | - |
| YES | 48 (30,8%) | 6 (12,5%) | 42 (87,5%) | 0,82 (0,30 – 2,25) | 0,702 |
| **Related to HIV** |  |  |  |  |  |
| **HIV carrier** |  |  |  |  |  |
| YES | 101 (66,4%) | 11 (10,9%) | 90 (89,1%) | 1,0 | - |
| NO | 51 (33,6%) | 11 (21,6%) | 40 (78,4%) | 2,25 (0,90 – 5,61) | 0,082 |
| **Use of Antiretroviral** |  |  |  |  |  |
| YES | 147 (93,6%) | 19 (12,9%) | 128 (87,1%) | 1,0 | - |
| NO | 10 (6,4%) | 3 (30,0%) | 7 (70,0%) | 2,89 (0,69 – 12,1) | 0,148 |
| **Current viral load** |  |  |  |  |  |
| undetectable | 117 (79,1%) | 15 (12,8%) | 102 (87,2%) | 1,0 | - |
| 50 a 100.000 | 29 (19,6%) | 6 (20,7%) | 78 (79,3%) | 1,77 (0,62 – 5,06) | 0,284 |
| > 100.000 copies | 2 (1,3%) | 0 (-) | 16 (100%) | Não calculado | - |
| **Current CD4** |  |  |  |  |  |
| > 200 | 138 (92,0%) | 20 (14,5%) | 118 (85,5%) | 1,0 | - |
| < 200 | 12 (8,0%) | 2 (16,7%) | 10 (83,3%) | 1,18 (0,24 – 5,78) | 0,838 |
| **CD4 Nadir** |  |  |  |  |  |
| > 200 | 94 (62,6%) | 13 (13,8%) | 81 (86,2%) | 1,0 | - |
| < 200 | 57 (37,4%) | 9 (15,8%) | 48 (84,2%) | 1,17 (0,46 – 2,94) | 0,741 |
| **Previous use of antibiotics** | |  |  |  |  |
| YES | 33 (21,0%) | 6 (18,2%) | 27 (81,8%) | 1,0 | - |
| NO | 124 (79,0%) | 16 (12,9%) | 108 (87,1%) | 0,67 (0,24 – 1,86) | 0,440 |

^a^ Statistically significant association (p < 0,05)
