# Supplementary material for: Prevalence of nasal colonization by methicillin-resistant Staphylococcus aureus in outpatients living with HIV/AIDS in a Referential Hospital of the Northeast of Brazil
Source: BMC Res Notes. 2018 Nov 6;11:794. doi: 10.1186/s13104-018-3899-z (PMC6219150; doi:10.1186/s13104-018-3899-z)
Supplement: Supplementary file 2 — Additional file 2: Table S2. Association of positivity by Staphylococcus aureus according to factors related to Comorbidities and co-infections of PLHA treated at an outpatient clinic at the HC/UFPE DIP Service. [file 13104_2018_3899_MOESM2_ESM.doc]

**Additional file 2**. Association of positivity by Staphylococcus aureus according to factors related to Comorbidities and co-infections of PLHA treated at an outpatient clinic at the HC / UFPE DIP Service.

| **Variables** | **All patients** | **Colonization by *Staphylococcus aureus*** | | **OR (IC 95%)** | **p-value** |
| --- | --- | --- | --- | --- | --- |
|  |  | **MRSA** | **MSSA** |  |  |
| **Comorbidades** |  |  |  |  |  |
| **Diabetes** |  |  |  |  |  |
| NO | 140 (91,5%) | 18 (12,9%) | 122 (87,1%) | 1,0 | - |
| YES | 13 (8,5%) | 4 (30,8%) | 9 (69,2%) | 3,01 (0,84 – 10,8) | 0,091 |
| **Chronic liver disease** |  |  |  |  |  |
| NO | 144 (92,9%) | 22 (15,3%) | 122 (84,7%) | 1,0 | - |
| YES | 11 (7,1%) | 0 (-) | 11 (100%) | Não calculado | 0,162 |
| **Hemodialysis** |  |  |  |  |  |
| NO | 151 (98,1%) | 21 (13,9%) | 130 (86,1%) | 1,0 | - |
| YES | 3 (1,9%) | 1 (33,3%) | 2 (66,7%) | 3,09 (0,27 – 35,6) | 0,365 |
| **Neoplasm** |  |  |  |  |  |
| NO | 150 (97,4%) | 22 (14,0%) | 129 (86,0%) | 1,0 | - |
| YES | 4 (2,6%) | 0 (-) | 4(100%) | Não calculado | 0,421 |
| **Sexually Transmitted Infections** |  |  |  |  |  |
| **Syphilis** |  |  |  |  |  |
| NO | 129 (82,2%) | 21 (16,3%) | 108 (83,7%) | 1,0 | - |
| YES | 28 (17,8%) | 1 (3,6%) | 27 (96,4%) | 0,19 (0,02 – 1,48) | 0,113 |
| **Genital herpes** |  |  |  |  |  |
| NO | 125 (79,6%) | 20 (16,0%) | 105 (84,0%) | 1,0 | - |
| YES | 32 (20,4%) | 2 (6,3%) | 67 (93,3%) | 0,35 (0,08 – 1,58) | 0,173 |
| **HPV infection** |  |  |  |  |  |
| NO | 146 (93,0%) | 20 (13,7%) | 126 (86,3%) | 1,0 | - |
| YES | 11 (7,0%) | 2 (18,2%) | 9 (81,8%) | 1,40 (0,28 – 6,95) | 0,681 |
| **Gonorrhea** |  |  |  |  |  |
| NO | 137 (87,3%) | 18 (13,1%) | 119 (86,9%) | 1,0 | - |
| YES | 20 (12,7%) | 4 (20,0%) | 16 (80,0%) | 1,65 (0,50 – 5,50) | 0,413 |

^a^ Statistically significant association (p < 0,05)
